# Risk factors for linezolid-associated thrombocytopenia in adult patients

**DOI:** 10.1007/s15010-014-0674-5

**Published:** 2014-08-14

**Authors:** B. Natsumoto, K. Yokota, F. Omata, K. Furukawa

**Affiliations:** 1 Department of Internal medicine, St. Luke’s International Hospital, 9-1 Akashi-Cho, Chuo-ku, Tokyo, 104-8560 Japan; 2 Department of Infectious Diseases, University of Kagawa, 1750-1 Ikedo, Mikichou, Kida-gunn, Kagawa 761-0793 Japan; 3 Center for Clinical Epidemiology, St. Luke’s Life Science Institute, 10-1 Akashi-Cho, Chuo-ku, Tokyo, 104-0044 Japan; 4 Department of Infectious Diseases, St. Luke’s Life Science Institute, 9-1 Akashi-Cho, Chuo-ku, Tokyo, 104-8560 Japan

**Keywords:** Linezolid, Thrombocytopenia, Dose, Adult

## Abstract

**Objectives:**

Thrombocytopenia (TP) is a common adverse effect of linezolid (LZD). However, risk factors for LZD-associated TP have been reported in Western patients with relatively heavy body weight. The aim of this study was to determine the risk factors for LZD-associated TP in Asian population.

**Materials and methods:**

A retrospective cohort study was conducted among 101 consecutive patients who received LZD therapy (1,200 mg/day) between July 2003 and December 2013 at a tertiary referral hospital in Tokyo, Japan. The patients with obvious other causes for TP were excluded. The information of target infectious disease, patients’ age, gender, body weight, body mass index, baseline serum creatinine (SCr), baseline platelet count, and treatment duration was collected retrospectively. TP was defined as ≥50 % decrease in platelet count from baseline. Bi- and multi-variate analyses were performed.

**Results:**

A total of 101 patients were included (mean age [SD] 64 [18]; male gender [%], 57 [56]). Median duration [range] of LZD therapy was 14 days [1–67]. LZD-associated TP was identified in 42 patients (42 %). For TP, adjusted odds ratio (OR) [95 % CI] of daily per kg dose (DPKD) and SCr was 1.14 [1.05–1.26] and 1.51 [1.01–2.50], respectively.

**Conclusions:**

Higher DPKD and elevated SCr are significantly associated with LZD-associated TP. These findings suggest that daily dose of LZD should be adjusted using body weight, as typically done in pediatrics, in adults as well. Renal function also should be considered for dose adjustment.

## Introduction

Linezolid (LZD) is an antimicrobial agent with a broad spectrum of activity against virtually all clinically important Gram-positive bacteria, including methicillin-resistant *Staphylococcus aureus* (MRSA) and vancomycin-resistant enterococci (VRE). An oxazolidinone, its mechanism of antimicrobial action is primarily bacteriostatic, inhibiting bacterial toxin production. Both the intravenous and oral formulations of LZD have nearly 100 % bioavailability due to its high water solubility and robust tissue penetration [1, 2]. LZD does not typically require dose adjustment by body weight (BW) in adults, though dose is determined by weight in pediatric patients [3, 4]. Thrombocytopenia (TP) is a common adverse effect in adult patients and the prevalence has been reported about 15–50 % with different definitions [5–9]. Prolonged treatment duration [9, 10], renal insufficiencies [1, 2, 9, 11–13], chronic liver disease [11], malignancy [14], previous vancomycin use [15], baseline platelet count [16], and lower BW [7, 8, 17] have been reported as possible risk factors for LZD-associated TP. However, most of previous studies have been conducted in Western patients with relatively heavy BW. The aim of this study was to identify the independent risk factors for LZD-associated TP in Asian population.

## Materials and methods

A retrospective cohort study was conducted among 101 consecutive patients who received LZD therapy (1,200 mg/day) for the first time between July 2003 and December 2013 at a tertiary referral hospital in Tokyo, Japan. Within the study period, 230 adults (age ≥ 20) have received LZD therapy. Those who had previously received LZD therapy (*n* = 85), with an acute DIC score ≥4 points (*n* = 24) [18], a hematological disorder (*n* = 15), or a definite diagnosis of prior drug-associated TP (*n* = 5) were excluded.

All patients received a total daily dose of 1,200 mg (600 mg, q12hr) regardless of BW. The information of target infectious disease, patients’ age, gender, BW, body mass index (BMI), baseline serum creatinine (SCr), baseline platelet count, and treatment duration was collected retrospectively. TP was defined as  ≥50 % platelet count decrease from baseline [19]. Baseline platelet count was defined as platelet count at initiation of LZD therapy. If there was no platelet data on the first day of LZD therapy, the closest previous platelet data prior to LZD therapy was used as baseline platelet count. Laboratory data were obtained between baseline and 14 days after discontinuation of LZD. Platelets were measured 2–3 times per week. Bi- and multi-variate analyses were performed.

### Statistical analysis

Fisher’s exact test was used for comparison of proportions, while Student’s *t* test was used for continuous variables. Bivariate and multivariate logistic regression analyses were subsequently conducted. Variables with *P* value less than 0.2 in bivariate analyses were principally added in multivariate analysis. Clinically relevant variables which have been previously reported to be associated with TP were also included in multivariate analysis.

All analyses including confidence intervals were two-sided, and *P* < 0.05 was considered statistically significant. All statistical analyses were performed using JMP® version 10 statistical software (SAS® Institute, Cary, NC).

## Results

A total of 101 patients were included in our analysis. Baseline characteristics are listed in Table 1. The patients’ mean age was 64 years, 56 % of them were male. Mean body weight was 57.3 kg. Patient with BMI less than 20 was 33 %. Mean (SD) daily per kg dose (DPKD) (mg/kg/day) of LZD was 21.39 (5.51). Its range (mg/kg/day) was [7.95–35.29]. Median duration [range] of LZD therapy was 14 days [1–67].

**Table 1:** Patients’ characteristics

Characteristics and underlying medical conditions	Value
Number of patients	101
Age (years)^a^, mean (SD)	64 (18)
Male, *n* (%)	57 (56)
BW (kg)^a^, mean (SD)	57.3 (17.3)
BMI (kg/m^2^)^a^, mean (SD)	23.02 (6.6)
BMI less than 20, *n* (%)	33 (33)
Treatment duration (days), median (range)	14 (1–67)
DPKD (1,200/BW (mg/kg)), mean (SD)	21.39 (5.51)
Serum creatinine (mg/dL)^a^, median (range)	0.87 (0.24–7.47)
eGFR (mL/min/1.73 m^2^)^a^, median (range)	61.58 (6.64–325.43)
CCr (mL/min)^a^, median (range)	61.29 (9.76–557.07)
Baseline Plt (×10^3^/μL)^a^, mean (SD)	266 (133)
Diagnosis	
Surgical site infection, *n* (%)	21 (20.8)
Cellulitis, *n* (%)	15 (14.9)
Urinary tract infection, *n* (%)	12 (11.9)
Artificial device infection^b^, *n* (%)	10 (9.9)
Osteomyelitis, *n* (%)	7 (6.9)
Pneumonia, *n* (%)	5 (5.0)
Pyothorax, *n* (%)	4 (4.0)
Infective endocarditis, *n* (%)	3 (3.0)
Epidural abscess, *n* (%)	2 (2.0)
Pyogenic arthritis, *n* (%)	2 (2.0)
Toxic shock syndrome, *n* (%)	2 (2.0)
Perforation of gastrointestinal tract, *n* (%)	2 (2.0)
Others, *n* (%)	14 (13.9)
Baseline disease	
Diabetes mellitus, *n* (%)	34 (33.7)
Hypertension, *n* (%)	58 (57.4)
Hyperlipidemia, *n* (%)	38 (37.6)

*BW* body weight, *BMI* body mass index, *DPKD* daily per kg dose, *eGFR* estimated glomerular filtration rate, *CCr* creatinine clearance

^a^Before linezolid administration

^b^Eight cases of intravascular device, one case of urine stent, one case of artificial breast (post expander-implant breast reconstruction)

LZD was mainly administrated for surgical infection, cellulitis, urinary tract infection, artificial device infection and osteomyelitis (Table 1). The main indication of LZD was definite or suspected MRSA infection. For these patients, LZD was chosen for following reasons: (1) side effects of other anti-MRSA antibiotics (49 %); (2) antibiotics failure (31 %); and (3) outpatient therapy (29 %). In 24 % of the total cases (MRSA 16.8 %, possible MRSA 6.9 %), LZD was administered due to the failure of other antimicrobial therapy. Patients with VRE infection were rare (3.9 %).

LZD-associated TP was found in 42 of 101 patients (42 %). The mean age, and DPKD were significantly higher in thrombocytopenic patients than in non-thrombocytopenic patients. The median creatinine clearance (CCr) was significantly lower in thrombocytopenic patients (Table 2). Platelet decreases of ≥50 % and final platelet counts of <150 × 10^3^/μL were identified in 31 % (31/101) of patients. TP with a final count of <100 × 10^3^/μL was seen in 26 % (26/101) of patients. Age, DPKD, BW and CCr were significantly associated with LZD-induced TP in bivariate analyses. For TP, adjusted odds ratio (OR) [95 % CI] of DPKD and SCr was 1.14 [1.05–1.26] and 1.51 [1.01–2.50], respectively (Table 3).

**Table 2:** Bivariate analyses

	Thrombocytopenic patients (*n* = 42)	Non-thrombocytopenic patients (*n* = 59)	*P* value
Age, mean (SD)	67.83 (15.59)	61.76 (19.05)	0.041**
Male, *n* (%)	22 (38.60)	35 (61.4)	0.49**
DPKD (1,200/BW) (mg/kg/day) mean (SD)	23.47 (5.10)	19.91 (5.35)	0.0011**
BW (kg), mean (SD)	53.64 (12.12)	64.75 (18.94)	0.0012**
SCr (mg/dL), median (range)	0.96 (0.24–7.47)	0.85 (0.24–3.93)	0.17*
^a^CCr (mL/min), median (range)	52.67 (9.76–153.73)	78.36 (13.63–557.07)	0.0299*
Baseline platelet (10^3^/mm^3^), mean (SD)	289.98 (20.37)	249.63 (17.19)	0.13**
Treatment duration (days), median (range)	14 (3–67)	14 (1–63)	0.36*

* Kruskal–Wallis test

** Student’s *t* test

^a^CCr (mL/min) = (140 − age) × weight/72 SCr, female times 0.85. Formula of Cockcroft

*SD* standard deviation, *DPKD* daily per kg dose, *BW* body weight, *SCr* serum creatinine, *CCr* creatinine clearance

**Table 3:** Bi-and multi-variate logistic regression

	Crude OR	*P* value	Adjusted OR (95 % CI)	*P* value
Age	1.02 (0.99–1.05)	0.09	1.01 (0.98–1.04)	0.44
SCr (mg/dL)	1.31 (0.90–2.07)	0.16	1.51 (1.01–2.50)	0.0457
DPKD (mg/kg/day)	1.14 (1.05–1.24)	0.001	1.14 (1.05–1.26)	0.0026
Treatment duration	1.01 (0.98–1.03)	0.71	1.01 (0.98–1.05)	0.52
Baseline platelet <200 × 10^3^/μL	0.81 (0.34–1.87)	0.62	1.00 (0.99–1.01)	0.24

*OR* odds ratio, *CI* confidence interval, *SCr* serum creatinine, *DPKD* daily per kg dose

**Fig. 1:**
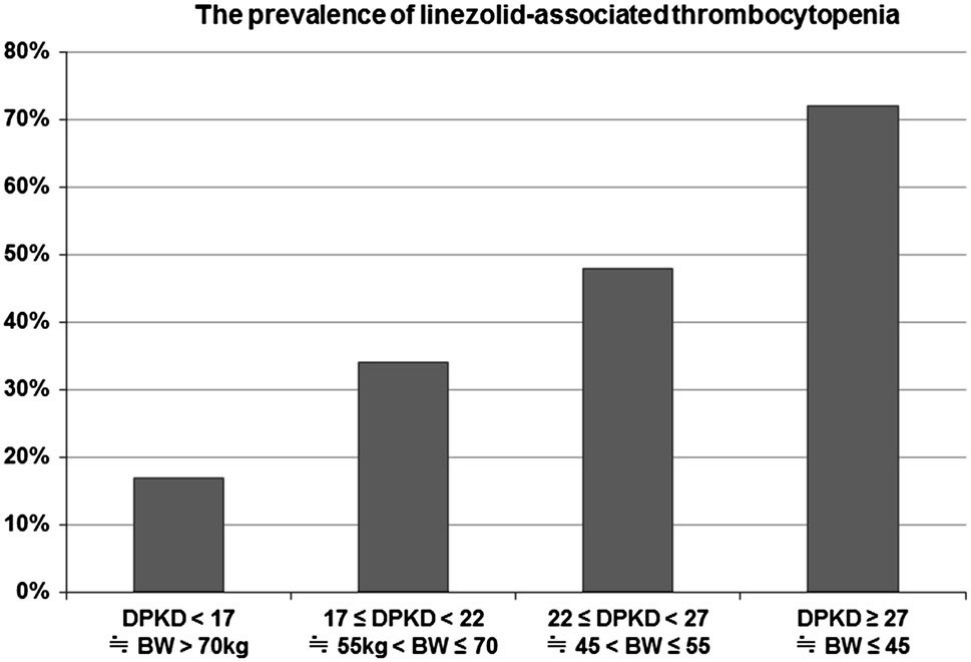
The prevalence of linezolid-associated thrombocytopenia stratified by daily per kg dose (DPKD) (mg/kg/day). The prevalence of thrombocytopenia is linearly associated with DPKD of linezolid. In 22 ≤ DPKD < 27, the prevalence of TP was 48 %. Its prevalence increased to 72 % in DPKD ≥ 27

When patients were categorized into four groups using three cut-off values (45, 55, 75 kg) of BW, the prevalence of TP in each group was 72 % (13/18), 48 % (12/25), 34 % (13/38), and 17 % (4/23) in the patients with DPKD ≥ 27 (BW ≤ 45 kg), 22 ≤ DPKD < 27 (45 kg < BW ≤ 55 kg), 17 ≤ DPKD < 22 (55 kg < BW ≤ 70 kg), DPKD < 17 (BW> 70 kg), respectively (Fig. 1).

## Discussion

Our study suggests that LZD-associated TP is associated with high DPKD and renal insufficiency.

The mechanism of LZD-associated TP is thought to be due to reversible myelosuppression [20]. Nonetheless, several case reports suggested that patients with LZD-associated TP retain adequate numbers of megakaryocytes in their bone marrow [21, 22]. Immune-mediated platelet destruction has been suggested based on a decreased rate of TP following immunoglobulin therapy [21]. Given multiple plausible mechanisms, the pathophysiology of LZD-associated TP remains controversial.

Niwa et al. [17], though using a different definition of TP at ≥25 % platelet decrease and final platelet count of <100 × 10^3^/μL, reported that DPKD ≥22 mg/kg and a baseline platelet count <200 × 10^3^/μL were significant risk factors for LZD-associated TP. Their study introduced the possibility of dose modification with linezolid [23]. In our study, we defined TP as a platelet decrease of ≥50 % as this level, while platelet count still in the normal range, may herald severe clinical problems, and requires active follow-up. Vanderschueren et al. [19] reported that drop in platelet count to <50 % of admission was associated with higher death rates in the ICU patients. Under this definition, LZD-associated TP was found in 42 of 101 patients (42 %) (Table 2). Considering the DPKD of 22 mg/kg that Niwa reported, when the patients were divided into four groups [DPKD ≥ 27 (≒BW ≤ 45 kg), 22 ≤ DPKD < 27 (≒45 kg < BW ≤ 55 kg), 17 ≤ DPKD < 22 (≒55 kg < BW ≤ 70 kg), DPKD < 17 (≒BW > 70 kg)], the prevalence of TP increased to 72 % in the group of DPKD ≥ 27 (≒ BW ≤ 45 kg) (Fig. 1) is an impressive result.

A recent randomized controlled study [5] reported that the incidence of LZD-associated TP (platelet count, <150 × 10^3^/μL if normal at baseline or 50 % decrease if low at baseline) was 16.3 %, the mean BW (SD) of subjects being 78.1 kg (23.3). This prevalence rate is similar to the prevalence of TP in the DPKD <17 category in our study.

According to LZD phase 3 trials in Japan, adverse events developed in 64.3 % (9/14) in those subjects with BW < 40 kg, and 53 % (44/83) in those with BW ≥ 40 kg [24]. These findings were similar to our results, in which the prevalence of TP was 70 % in patient ≤45 kg.

In pediatric patients, especially those with lower BW, the daily dose of LZD requires adjustment by BW. We think that it is similarly reasonable to suggest weight-based dosing for LZD even in relatively lower weight adults.

Elevated SCr was also independently associated with LZD-associated TP. A previous phase 3 trial [24] showed that the pharmacokinetics of LZD is not influenced by age, hepatic function, or renal function (CCr > 30). Moreover, in serial oral administration tests for patients with end-stage renal disease, plasma LZD concentrations were not influenced by renal function [24]. LZD is metabolized by non-enzymatic chemical oxidation and 30–40 % is excreted unchanged in the urine [25–30]. It is thought that LZD is not metabolized by cytochrome P450, as it shows none of the induction or inhibitory effects associated with various human cytochrome P450 enzyme activities. However, after coming to market, the association between LZD-associated adverse events (including TP) and renal insufficiency [1, 2, 12], chronic liver disease [12], prolonged administration [11], malignancy [15] and previous vancomycin use [16] have been reported. In particular, renal insufficiency has been reported frequently as a risk factor of LZD-associated TP in recent studies [1, 2, 31, 32]. Our results corroborate these findings. Prolonged treatment duration (TD) of LZD was reported as the main risk factor of LZD-associated TP [16, 33]. However, our study showed no association between TD and TP.

Area under the blood concentration–time curve (AUC) value of LZD has been reported to be higher in subject older than 80 years and BW less than 40 [24]. The mean AUC value (SD) of these subjects was 811.3 (280.7) μg h/mL. This value is 3.7 times of the subjects with age <80 and BW ≥ 40 [217.6 (129.9) μg h/mL] [24]. On the safety of high-exposure examination, adverse event prevalence was found in 7 of 11 subject (63.6 %) in the high-exposure subjects (AUC ≥ 800 μg h/mL), 41 of 80 (51.3 %) in the non-high-exposure subjects (AUC < 800 μg h/mL). Nukui et al. [32] reported that high plasma LZD trough concentration is a risk factor for TP. Dong et al. [7] reported the minimum trough level (*C*_min_) of linezolid was significantly higher in patients with TP than in those without TP (8.81 mg/L [1.98–37.54] vs. 2.88 mg/L [0.35–8.78], *P* < 0.0001). Matsumoto et al. [34] reported that the trough concentration of LZD [mg/L] was 14.4–35.6 versus (vs.) 6.9–7.2 and the area under the plasma linezolid concentration–time curve for 24 h (AUC24 h) [mg h/L] was 513.1–994.6 vs. 294.3–323.6 in the thrombocytopenic vs. non-thrombocytopenic patients. In addition, several recent Japanese studies have discussed the relationship between LZD blood concentration and TP [35–37]. As mentioned above, the fact that AUC value of LZD is relatively high in low BW subjects has been previously demonstrated in the phase 3 clinical trials in Japan [24]. Our results strongly suggest that high DPKD and elevated SCr are independently associated with LZD-associated TP. Therefore, we assume that higher DPKD and renal dysfunction are related to TP via higher serum LZD concentrations.

This is the first study suggesting a DPKD-dependent linear association between LZD and TP by categorizing DPKD into four groups. As this is a single center study among Japanese, our findings warrant external validation.

In conclusion, both higher DPKD and elevated SCr are significant risk factors for LZD-associated TP. As is done in pediatric patients, the daily dose of LZD should be adjusted by BW in adults as well. Renal function also should be considered for dose adjustment.

## References

[1] Lin YH, Wu VC, Tsai IJ (2006). High frequency of linezolid-associated thrombocytopenia among patients with renal insufficiency. Int J Antimicrob Agents.

[2] Wu VC, Wang YT, Wang CY (2006). High frequency of linezolid-associated thrombocytopenia and anemia among patients with end-stage renal disease. Clin Infect Dis.

[3] Jungbluth GL, Welshman IR, Hopkins NK (2003). Linezolid pharmacokinetics in pediatric patients: an overview. Pediatr Infect Dis..

[4] Chiappini E, Conti C, Galli L (2010). Clinical efficacy and tolerability of linezolid in pediatric patients: a systematic review. Clin Ther.

[5] Wunderink RG, Niederman MS, Kollef MH (2012). Linezolid in methicillin-resistant *Staphylococcus aureus* nosocomial pneumonia: a randomized. Controlled Study. Clin Infect Dis..

[6] Bi LQ, Zhou J, Huang M (2013). Efficacy of linezolid on gram-positive bacterial infection in elderly patients and the risk factors associated with thrombocytopenia. Pak J Med Sci..

[7] Dong HY, Xie J, Chen LH (2014). Therapeutic drug monitoring and receiver operating characteristic curve prediction may reduce the development of linezolid-associated thrombocytopenia in critically ill patients. Eur J Clin Microbiol Infect Dis.

[8] Chen C, Guo DH, Cao X (2012). Risk factors for thrombocytopenia in adult Chinese patients receiving linezolid therapy. Curr Ther Res Clin Exp.

[9] Hirano R1, Sakamoto Y, Tachibana N, et al. Retrospective analysis of the risk factors for linezolid-induced thrombocytopenia in adult Japanese patients. Int J Clin Pharm. 2014;36:795–9. 10.1007/s11096-014-9961-6 24913359

[10] Attassi K, Hershberger E, Alam R (2002). Thrombocytopenia associated with linezolid therapy. Clin Infect Dis.

[11] Sasaki T, Takane H, Ogawa K (2011). Population pharmacokinetic and pharmacodynamic analysis of linezolid and a hematologic side effect, thrombocytopenia, in Japanese patients. Antimicrob Agents Chemother.

[12] Cossu AP, Musu M, Mura P (2014). Linezolid-induced thrombocytopenia in impaired renal function: is it time for a dose adjustment? a case report and review of literature. Eur J Clin Pharmacol.

[13] Soriano A, Ortega M, García S (2007). Comparative study of the effects of pyridoxine, rifampin, and renal function on hematological adverse events induced by linezolid. Antimicrob Agents Chemother.

[14] Smith PF, Birmingham MC, Noskin GA (2003). Safety, efficacy and pharmacokinetics of linezolid for treatment of resistant Gram-positive infections in cancer patients with neutropenia. Ann Oncol.

[15] Rao N, Ziran BH, Wagener MM (2004). Similar hematologic effects of long-term linezolid and vancomycin therapy in a prospective observational study of patients with orthopedic infections. Clin Infect Dis.

[16] Grau S, Morales-Molina JA, Mateu-de Antonio J (2005). Linezolid: low pre-treatment platelet values could increase the risk of thrombocytopenia. J Antimicrob Chemother.

[17] Niwa T, Suzuki A, Sakakibara S (2009). Retrospective cohort chart review study of factors associated with the development of thrombocytopenia in adult Japanese patients who received intravenous linezolid therapy. Clin Ther.

[18] Gando S, Iba T, Eguchi Y (2006). A multicenter, prospective validation of disseminated intravascular coagulation diagnostic criteria for critically ill patients: comparing current criteria. Crit Care Med.

[19] Vanderschueren S, De Weerdt A, Malbrain M, et al. Thrombocytopenia and prognosis in intensive care. Crit Care Med. 2000;28:1871–6. 10.1097/00003246-200006000-00031 10890635

[20] Senneville E, Legout L, Valette M (2004). Risk factors for anaemia in patients on prolonged linezolid therapy for chronic osteomyelitis: a case-control study. J Antimicrob Chemother.

[21] Bernstein WB, Trotta RF, Rector JT (2003). Mechanisms for linezolid-induced anemia and thrombocytopenia. Ann Pharmacother.

[22] Ebeling F, Helminen P, Anttila VJ (2009). Appearance of ring sideroblasts in bone marrow during linezolid therapy. Scand J Infect Dis.

[23] Niwa T, Watanabe T, Suzuki A (2014). Reduction of linezolid-associated thrombocytopenia by the dose adjustment based on the risk factors such as basal platelet count and body weight. Diagn Microbiol Infect Dis.

[24] Linezolid application document for the manufacture and sales approval in Japan. PMDA website http://www.info.pmda.go.jp/downfiles/ph/PDF/671450_6249401A1025_4_11.pdf

[25] Slatter JG, Stalker DJ, Feenstra KL (2001). Pharmacokinetics, metabolism, and excretion of linezolid following an oral dose of [14C]linezolid to healthy human subjects. Drug Metab Dispos.

[26] Abe S, Chiba K, Cirincione B (2009). Population pharmacokinetic analysis of linezolid in patients with infectious disease: application to lower body weight and elderly patients. J Clin Pharmacol.

[27] McGee B, Dietze R, Hadad DJ (2009). Population pharmacokinetics of linezolid in adults with pulmonary tuberculosis. Antimicrob Agents Chemother.

[28] Meagher AK, Forrest A, Rayner CR (2003). Population pharmacokinetics of linezolid in patients treated in a compassionate-use program. Antimicrob Agents Chemother.

[29] Plock N, Buerger C, Joukhadar C (2007). Does linezolid inhibit its own metabolism? population pharmacokinetics as a tool to explain the observed nonlinearity in both healthy volunteers and septic patients. Drug Metab Dispos.

[30] Whitehouse T, Cepeda JA, Shulman R (2005). Pharmacokinetic studies of linezolid and teicoplanin in the critically ill. J Antimicrob Chemother.

[31] Matsumoto K, Takeda Y, Takeshita A (2009). Renal function as a predictor of linezolid-induced thrombocytopenia. Int J Antimicrob Agents.

[32] Nukui Y, Hatakeyama S, Okamoto K (2013). High plasma linezolid concentration and impaired renal function affect development of linezolid-induced thrombocytopenia. J Antimicrobial Chemotherapy..

[33] Gerson SL, Kaplan SL, Bruss JB (2002). Hematologic effects of linezolid: summary of clinical experience. Antimicrob Agents Chemother.

[34] Matsumoto K, Takeshita A, Ikawa K (2010). Higher linezolid exposure and higher frequency of thrombocytopenia in patients with renal dysfunction. Int J Antimicrob Agents.

[35] Tsuji Y, Hiraki Y, Matsumoto K (2011). Thrombocytopenia and anemia caused by a persistent high linezolid concentration in patients with renal dysfunction. J Infect Chemother..

[36] Hiraki Y, Tsuji Y, Matsumoto K (2011). Influence of linezolid clearance on the induction of thrombocytopenia and reduction of hemoglobin. Am J Med Sci.

[37] Hiraki Y, Tsuji Y, Hiraike M (2012). Correlation between serum linezolid concentration and the development of thrombocytopenia. Scand J Infect Dis.

